# Comparative acetylome analysis reveals the potential roles of lysine acetylation for DON biosynthesis in *Fusarium graminearum*

**DOI:** 10.1186/s12864-019-6227-7

**Published:** 2019-11-12

**Authors:** Shanyue Zhou, Chunlan Wu

**Affiliations:** 1 College of Plant Health and Medicine, The Key Lab of Integrated Crop Pests Management of Shandong Province, Qingdao Agricultural University, No. 700 Changcheng Road, Chengyang, Qingdao, 266109 Shandong China; 2 State Key Laboratory of Crop Stress Biology for Arid Aeras, Northwest A&F University, Yangling, 712100 Shaanxi China

**Keywords:** *Fusarium graminearum*, Deoxynivalenol, Lysine acetylation, Acetylome

## Abstract

**Background:**

*Fusarium graminearum* is a destructive fungal pathogen of wheat, barley and other small grain cereals. During plant infection, the pathogen produces trichothecene mycotoxin deoxynivalenol (DON), which is harmful to human and livestock. *FgGCN*5 encodes a GCN5 acetyltransferase. The gene deletion mutant *Fggcn5* failed to produce DON. We assumed that lysine acetylation might play a key regulatory role in DON biosynthesis in the fungus.

**Results:**

In this study, the acetylome comparison between *Fggcn*5 mutant and wild-type strain PH-1 was performed by using affinity enrichment and high resolution LC-MS/MS analysis. Totally, 1875 acetylated proteins were identified in *Fggcn*5 mutant and PH-1. Among them, 224 and 267 acetylated proteins were identified exclusively in *Fggcn*5 mutant and PH-1, respectively. Moreover, 95 differentially acetylated proteins were detected at a significantly different level in the gene deletion mutant:43 were up-regulated and 52 were down-regulated. GO enrichment and KEGG-pathways enrichment analyses revealed that acetylation plays a key role in metabolism process in *F. graminearum*.

**Conclusions:**

Seeing that the gens playing critical roles in DON biosynthesis either in *Fggcn5* mutant or PH-1. Therefore, we can draw the conclusion that the regulatory roles of lysine acetylation in DON biosynthesis in *F. graminearum* results from the positive and negative regulation of the related genes. The study would be a foundation to insight into the regulatory mechanism of lysine acetylation on DON biosynthesis*.*

## Background

*Fusarium graminearum* is a disastrous fungal pathogen which causes Fusarium head blight (FHB) on wheat, barley and other small grain cereals [1, 2]. In addition to the severe yield loss and quality damage, the pathogen produces trichothecene-type mycotoxins, such as deoxynivalenol (DON) in the infected tissue. DON is a secondary metabolite, which contributes to the spread of the fungus in the spikelet and contaminates cereal grains and cereal-based products, resulting in a threat to the health of human and livestock [3, 4].

Lysine acetylation is a conserved post-translational modification (PTM) of proteins occurring both in eukaryotes and prokaryotes. The modification consists of two reversible reactions: the acetylation, in which the acetyl-groups were added to the lysine residues of target protein by lysine acetyltransferase (KAT); in contrast, the deacetylation is a reversed process to remove the acetyl-groups from the acetylated proteins by lysine deacetylase (KDAC) [5, 6]. The balance of acetylation/deacetylation status of proteins is dynamically regulated by KATs and KDACs in order to achieve their proper roles during numerous cellular processes such as cell morphology, metabolic pathways, protein synthesis [7–9]. The acetylation was first identified in histone proteins, whose acetylated form is responsible for the structure remodeling of the chromatin and activation of genes expression [10, 11]. In recent years, the protein acetylation has been widely studied by using advanced mass spectrometry based proteomics tool. Global analyses of acetylome have been successfully performed in plants [12, 13], fungi [14, 15], and prokaryotes [16, 17], revealing that acetylation contributes to diverse protein functions in living cells, including protein localization, enzymatic activity, protein-protein and protein-nucleic acids interaction [18–20].

The lysine acetylation also plays a crucial role in regulating central metabolism as the extensively acetylated enzymes responsible for metabolism have been found in both eukaryotes and prokaryotes [9, 17, 21]. For instance, most enzymes involved in glycolysis, the tricarboxylic (TCA) cycle, gluconeogenesis, the urea cycle, and fatty acid metabolism were acetylated in human liver tissue [22]. A global acetylome analysis in *Salmonella enterica* revealed that about 90% of the enzymes of central metabolism were found to be acetylated [8]. In addition, the protein acetylation is also involved in the secondary metabolism process, such as nonribosomal peptide synthesis, hydroxamate siderophore and phosphinic acid products biosynthesis [20].

The gene *FgGCN5* (FGRAMPH1_01T00753) in *F. graminearum* PH-1 encodes a GCN5 acetyltransferase. The most attractive defect of the gene deletion mutant is the functional block in DON biosynthesis [23], indicating that the gene plays a crucial role in producing DON in the fungus. To reveal the potential roles of lysine acetylation in DON biosynthesis, we performed a global acetylome comparison between the gene deletion mutant *Fggcn5* and the wild-type strain PH-1. Totally, 2626 acetylated lysine sites in 1875 proteins were identified in *Fggcn5* mutant and PH-1.

## Results and discussion

### Difference of the acetylated proteins between the wild type and Fggcn5 deletion mutant

**Fig. 1:**
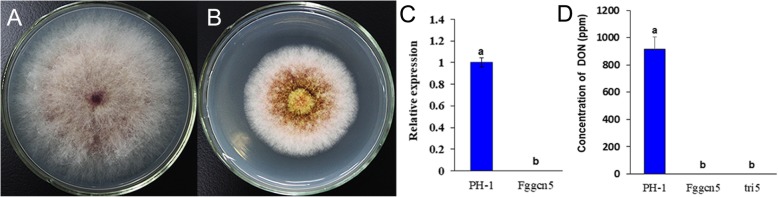
Colony and DON production in *Fggcn5* mutant. **a** Colony of the wild-type strain PH-1. **b** Colony of the *Fggcn5* mutant on PDA. **c** Expression of the *FgGCN5* gene in PH-1 and *Fggcn5* mutant. **d** DON production in *Fggcn5* mutant, PH-1 and negative control *tri*5 mutant

The predicted gene in the *F. graminearum* genome, FGRAMPH1_01T00753, is orthologous to yeast *GCN5* and its lysine acetyltransferase activity was confirmed in a previous study [23]. To gain insights into the possible acetylome regulated by *FgGCN5* in *F. graminearum*, we generated the gene deletion construct by the split-marker approach [24] and transformed it into the wild-type strain PH-1. As shown in Fig. 1, the *Fggcn5* deletion mutant significantly reduced hyphae growth (growth rate is 53.45% of PH-1), and failed to produce DON.

To identify proteins acetylated by FgGCN5, total proteins were isolated from PH-1 and *Fggcn5* mutant. After digestion with trypsin, lysine-acetylated peptides were enriched with the anti-acetyl-lysine antibody and analyzed with LC-MS/MS as described [25]. A total of 2626 lysine acetylation sites (Additional file 1: Table S1) were identified in 1875 proteins from PH-1 and *Fggcn5* mutant (Additional file 2: Table S2). Among them, 95 proteins were differentially acetylated at a significant level of Ratio > + /− 2 (*p* < 0.05) in the *Fggcn5* deletion mutant in comparison with PH-1. 43 proteins were up-regulated, 52 were down-regulated in the mutant (Additional file 3: Table S3). It is possible that the acetylation down-regulated proteins in the *Fggcn5* mutant function in a positive slight role in the DON biosynthesis, while the up-regulated proteins play the opposite role.

In comparison with the *Fggcn5* mutant, 274 acetylated lysine sites of the 267 proteins were identified exclusively in the wild-type strain PH-1 (Additional file 4: Table S4). Some proteins likely to be acetylated by FgGCN5 have been functionally characterized (Table 1). We also identified 226 acetylated lysine sites in 224 proteins that only present in the *Fggcn5* mutant. Deletion in *FgGCN5* somehow stimulated acetylation on these proteins in *F. graminearum*. It is possible that other lysine acetyltransferases were activated to acetylate these proteins in the absence of *FgGCN5*. Some of these proteins have been functionally characterized (Table 2).

**Table 1:** Acetylated proteins specially detected in wild type strain PH-1

Protein	Gene number	Annotation	Function	Reference
FgFkbp12	FGSG_09690	Rapamycin binding protein	Rapamycin toxicity	[26]
FaTUA1	FGSG_00639	α-tubulin	Virulence, hyphae growth	[27]
GzOB031	FGSG_08737	Transcription factor	Virulence	[28]
GzBrom002	FGSG_06291	Transcription factor	DON, virulence, sexual and asexual	[28]
FGSG_10825	FGSG_10825	Homocysteine transferase	DON, virulence and development	[29]
FGK3	FGSG_07329	Glycogen synthase kinase	DON, virulence and development	[30]
PKR	FGSG_09908	Protein kinase	DON, virulence, sexual and asexual	[31]
FCA6	FGSG_02974	Peroxidase	Peroxidase activities	[32]

**Table 2:** Acetylated proteins specially detected in *Fggcn5* mutant

Protein	Gene number	Annotation	Function	Reference
GzHMG002	FGSG_00385	Transcription factor	DON, virulence and development	[28]
GzCCHC011	FGSG_10716	Transcription factor	DON, virulence and development	[28]
GzZC230	FGSG_07133	Transcription factor	DON, virulence	[28]
FgHXK1	FGSG_00500	Hexokinase	DON, virulence and development	[33]
FgSKN7	FGSG_06359	Transcription factor	DON, virulence and development	[34]
FaMyo2B	FGSG_07469	Myosin protein	Virulence and development	[35]
FgArb1	FGSG_04181	ABC transporter	DON, virulence and development	[36]
FgATG8	FGSG_10740	Autophagy protein	Sexual and asexual development	[37]
CDC2B	FGSG_03132	Kinase	Asexual and vegetative growth	[38]
TRI15	FGSG_11205	zinc-finger protein	DON	[39]

The abundance of acetylated proteins detected in this study indicated that lysine acetylation is a common protein modification in *F. graminearum* similar to the observations in other living organisms [12–15]. Approximately 14.24% of the acetylated proteins identified in this study were only detected in the wild type strain and are potential targets of *FgGCN5* lysine acetyltransferase.

### Functional annotation and enrichment analysis of the proteins differentially acetylated in PH-1 and the Fggcn5 mutant

**Fig. 2:**
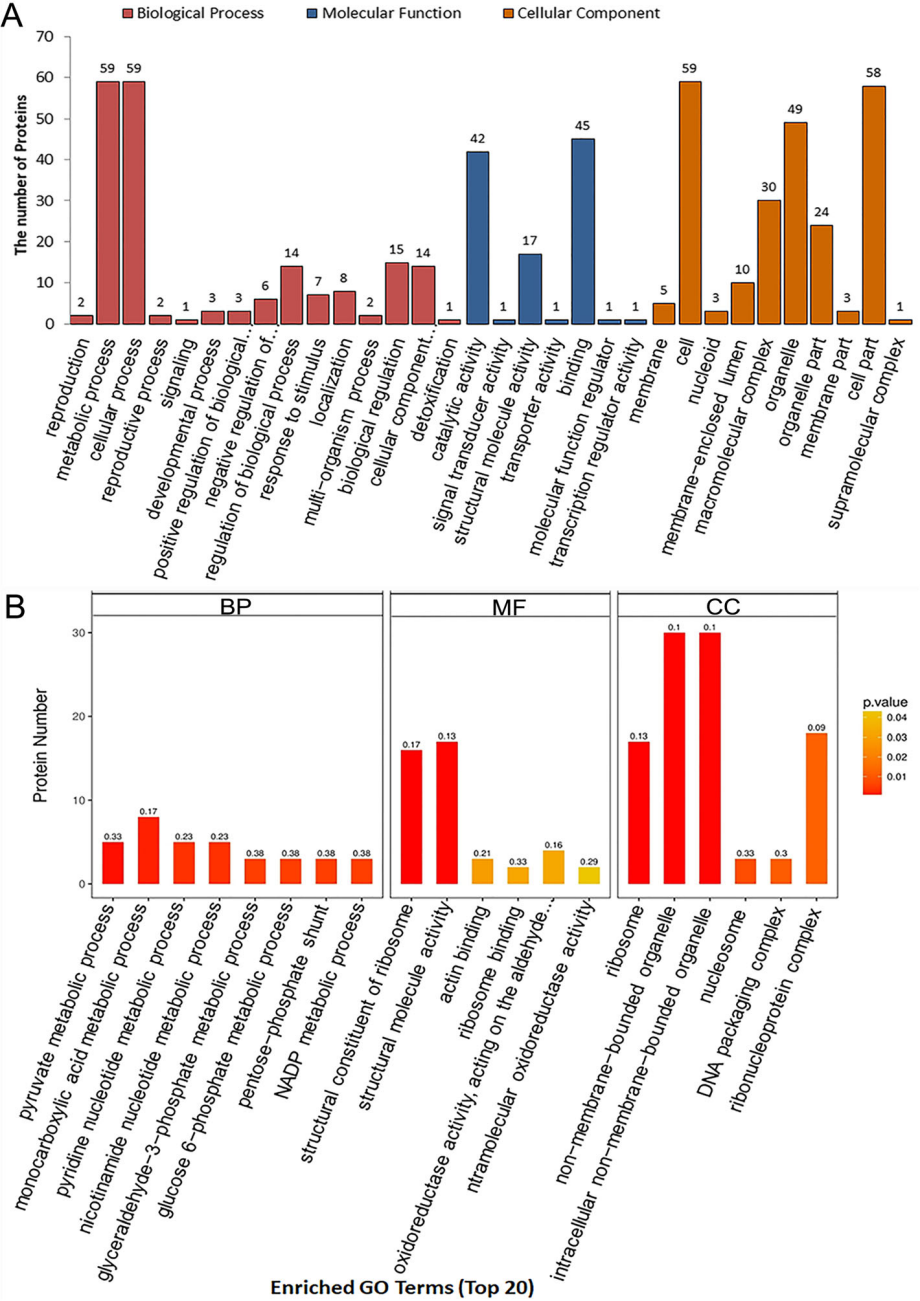
GO and GO enrichment of the identified acetylated proteins. **a** GO analysis of the identified acetylated proteins. The proteins were classified according to their biological processes, molecular functions and cellular compartments. Numbers of proteins in different classification were shown on top of the columns. **b** GO enrichment analysis of the identified acetylated proteins

To determine the functions of acetylated proteins, we analyzed GO annotation and classified the identified proteins according to their biological processes, molecular functions and cellular compartments. In the GO biological processes, 59 proteins were involved in metabolic processes, 59 in cellular processes, 15 in biological regulation, and 14 in the regulation of biological processes and cellular compartment organization or biogenesis. According to GO molecular function category, 42 proteins were involved in catalytic activities, 45 in binding activities, and 17 in structural molecular activity. With respect to the cellular compartments on level 2, 59 proteins were cell proteins, 58 were cell part proteins, 49 were organelle proteins, 30 were macromolecular-complex proteins, 24 were organelle part proteins, and 10 were membrane-enclosed lumen proteins (Fig. 2a).

Furthermore, the GO enrichment analysis was performed to identify the biological processes and molecular functions of the acetylated proteins (Fig. 2b, Additional file 5: Table S5). The results showed that the acetylated proteins identified in this study were significantly enriched in several GO biological processes, including monocarboxylic acid metabolism, pyridine nucleotide metabolism, nicotinamide nucleotide metabolism, pyruvate metabolic process, glucose 6-phosphate metabolic process, glyceraldehyde-3- phosphate metabolic process, and NADP metabolic process. In the GO molecular functions, most of the acetylated proteins were significantly enriched in structural molecular activity, structural constituent of ribosome, oxidoreductase activity. From the GO cellular compartment categories, we found that a great proportion of the identified acetylated proteins were in intracellular non-membrane-bounded organelles, ribonucleoprotein complexes, and ribosome.

**Fig. 3:**
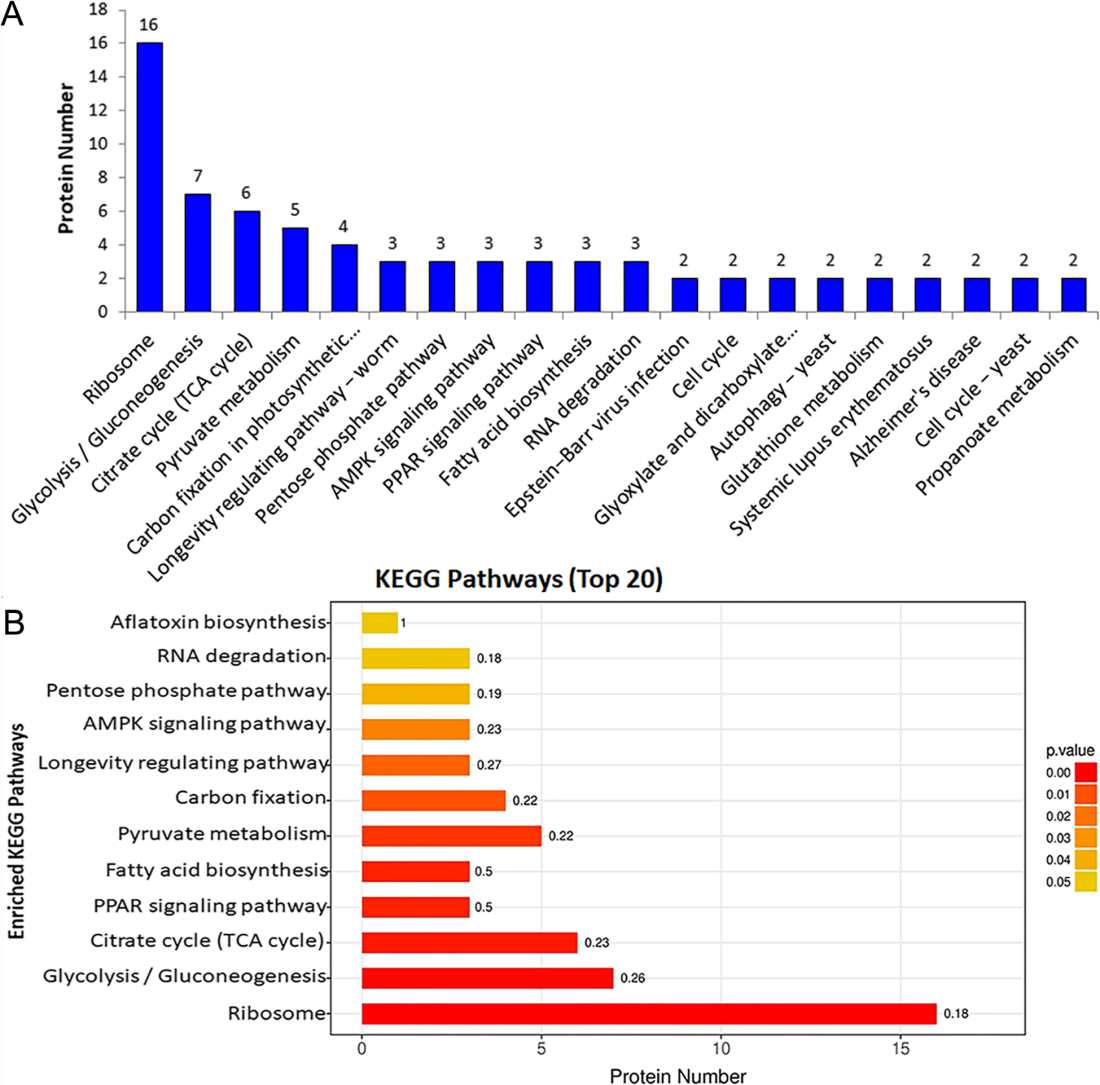
KEGG pathway and KEGG pathway enrichment of the identified acetylated proteins. **a** KEGG pathway analysis of the proteins involved in and the numbers of proteins in different pathways were shown on top of the columns. **b** KEGG pathway enrichment analysis of the identified acetylated proteins

The KEGG-pathways in which the acetylated proteins involved were analyzed (Fig. 3a). The results revealed that proteins were enriched in several conserved pathways such as ribosomes, glycolysis/gluconeogenesis and citrate cycle (TCA cycle) (Fig. 3b, Additional file 6: Table S6). Moreover, the KEGG-pathways enriched in fatty acid biosynthesis, pyruvate metabolism suggested that the acetylation play important roles in cell metabolic processes. It is in agreement with the well-established conclusion that lysine acetylation plays key roles in regulation of the metabolic pathways [8, 17, 40]. Further, the acetylate form of the enzymes involved in the acetyl-CoA synthesis, such as pyruvate dehydrogenase E2 (FG04171.1) and long-chain acyl-CoA synthetase (FG08543.1) were detected only in PH-1 but not in the *Fggcn5* mutant. It should be noted that the acetyl-CoA is essential for the DON synthesis. Therefore, the GO and KEGG enrichment provided powerful evidence for the role of acetylation in DON synthesis.

### Protein-protein interaction network analysis

**Fig. 4:**
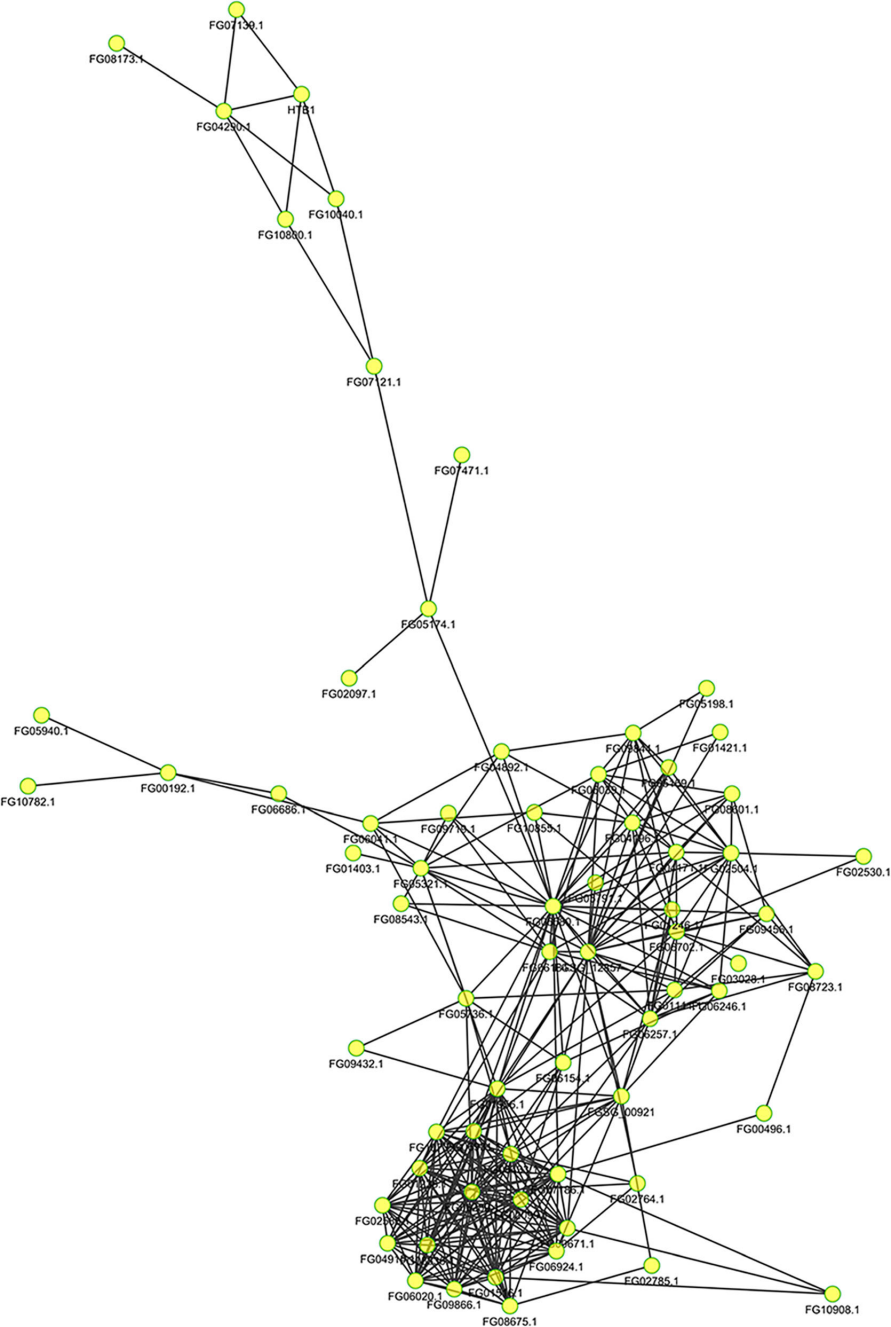
Protein-protein interaction network of the acetylated proteins. 93 acetylated proteins were mapped into the protein-protein interaction network using STRING database

To better understand the cellular processes regulated by lysine acetylation, the protein-protein interaction network was predicted as described [41]. In total, 93 acetylated proteins were mapped into the protein-protein interaction network (Additional file 7: Table S7). As shown in Fig. 4, the network overviews the physical and functional interactions of the acetylated proteins in *F. graminearum*. Obviously, the ribosome-associated proteins, metabolism-associated proteins, especially proteins involved in the citrate cycle were specifically enriched. These findings suggest that acetylation plays a key role in protein biosynthesis and central metabolic processes. Interestingly, the core component of nucleosome Histone H3 and Histone H2B were involved in the network. The H3-interacting protein (FGSG-08173) is predicted to be homologous to the Pim1 Ser/Thr protein kinase, which plays an important role in signal transduction related to energy metabolism and cell proliferation and survival in humans [42, 43]. Another H3-interacting protein (FGSG_10,040) is predicted to encode FACT complex subunit SPT16, which was demonstrated to participate in specific regulation on genes transcription in yeast [44]. It was well demonstrated that acetylation of histone H3 by FgGCN5 is directly related to DON biosynthesis [45]. It is possible that histone H3 was co-regulated by FgGCN5 and kinase FGSG-08173. The acetylation and phosphorylation of histone H3 contributes to the activation or inactivation of FGSG-10040 in the FACT complex, which in turn affects the re-organization of nucleosomes. As a result, the transcription of genes involved in the DON production were initiated or blocked.

### Proteins acetylated in PH-1 involved in DON biosynthesis

Since the *Fggcn5* gene deletion mutant was defective in DON biosynthesis, lysine acetylation manipulated by FgGCN5 likely plays important regulatory roles in DON biosynthesis in *F. graminearum*. In this study, we found that some proteins involved in DON production are the potential acetylation targets of FgGCN5.

*GzBrom002* (FGSG_06291) encoding a transcription factor plays essential roles in DON production, virulence, asexual and sexual reproduction. The gene deletion mutant *GzBrom002* completely lost virulence on wheat, ability in DON production and asexual and sexual spores production [28]. A Homocysteine transferase gene (FGSG_10825) is also multifunctional in *F. graminearum*. Phenotype assays showed that the virulence and DON production were reduced in the gene deletion mutant Moreover, the mutant failed to produce perithecia and aerial mycelia [29]. Another gene *FGK*3 (FGSG-07329), encodes a glycogen synthase kinase orthologous to mammalian *GSK*3. The gene deletion mutant Δ*fgk*3 is defective in DON production [30]. FgGCN5 might positively regulate DON biosynthesis through acetylating these proteins.

It has been well demonstrated that cAMP- dependent protein kinase (PKA) plays critical roles in DON biosynthesis in *F. graminearum* [46, 47]. In this study, PKR (FGSG_09908), the regulatory subunit of PKA, was found to be acetylated in PH-1 but not in the *Fggcn5* mutant. The result indicates that the PKR may be one of the substrates of FgGCN5 acetyltransferase in *F. graminearum*. However, PKR acts as a negative factor in DON production as the DON content was increased in the gene deletion mutant of PKR [31]. This suggests that the negative effect of PRK on DON biosynthesis may be limited by FgGCN5 through lysine acetylation.

### Proteins acetylated in Fggcn5 mutant are associated with DON production

Proteins acetylated specifically in *Fggcn5* mutant were identified as well, suggesting that the proteins are targets of other KATs rather than *FgGCN5.* Interestingly, some proteins were proved to be associated with DON biosynthesis.

It is well known on the functions of some *TRI* genes in DON biosynthesis. In this study, the acetylated TRI15 (FGSG_11205) was detected only in the *Fggcn5* mutant. *TRI*15, encoding a Cys2-His2 zinc finger protein, acts as a negative regulator of the trichothecene biosynthetic genes [39, 48]. It is likely that TRI15 is activated by other KATs and thereby plays a negative role in DON production in *Fggcn5* mutant.

Additionally*, FgHXK*1(FGSG_00500) encodes a rate-limiting enzyme in DON biosynthesis. DON production was severely inhibited in the gene deletion mutant. Moreover, the *ΔFgHXK*1 mutant is nonpathogenic on wheat, defective in hyphae growth and conidiation [33]. Some transcription factors identified in this study were also characterized to play key roles in DON production and pathogen virulence including *GzHMG002* (FGSG_00385), GzCCHC011 (FGSG_10716) and GzZC230 (FGSG_07133) [28]. Recently, a ATP-binding cassette (ABC) transporte FgArb1 (FGSG_04181) was proved to function in pathogenesis and DON production in *F. graminearum*, as the virulence and DON production were dramatically reduced in the gene deletion mutant [36]. It is likely that acetylation of these proteins by other KATs in the absence of FgGCN5 leads to the inactivation of the genes, and finally leads to the inhibition of the DON production in *Fggcn5* mutant.

## Conclusions

In summary, the acetylome comparison between *Fggcn5* mutant and PH-1 was performed by high throughput proteomics analysis. The differentially acetylated proteins were identified. Our results indicate that genes play critical roles in DON production in Fggcn5 mutant or PH-1. Therefore, we can draw the conclusion that the DON biosynthesis in *F. graminearum* was properly regulated by lysine acetylation both in positive and negative ways. The study would be a foundation to insight into the regulatory mechanism of lysine acetylation on DON production*.*

## Methods

### Generation of *Fggcn*5 mutant

The gene deletion mutant *Fggcn*5 was generated with the split-marker approach [24]. For the mutant, a 790 bp upstream and an 820 bp downstream flanking DNA sequences of *FgGCN5* were amplified with primers 280-1F/280-2R and 280-3F/280-4R, respectively (Table 3). The PCR products were then connected to the hygromycin phosphotransferase (*hph*) fragments amplified with the primers HYG-F/HY-R and YG-F/HYG-R (Table 3) by overlapping PCR. And the resulting PCR products were then transformed into protoplasts of PH-1 following a described method [49]. For protoplasts production, the conidia of PH-1 were incubated in YEPD (yeast extract 3 g, peptone 10 g, dextrose 20 g per liter) broth at 25 °C. After incubation for 12 h, the mycelia were harvested by filtration with sterile microcloth and digested in lysing buffer (25 mg/mL driselase and 5 mg/mL lysing enzyme in 1.2 M KCL) for 2 h. After filtration through a 30 μm Nitex nylon membrane, the digestion mixture was centrifugated at room temperature. The protoplasts were gently resuspended in STC buffer (20% sucrose, 10 mM tris pH 8.0, 50 mM CaCL_2_). The PCR products were added into the protoplast solution and PTC (40% PEG8000 in STC) was added subsequently. The solution mixture was added into TB3 (3 g Yeast extract, 3 g Casamino acids, 20% sucrose in 1 l) containing Hygromycin B to 300 μg/mL and pour plates. Then the plates were incubated at 25 °C. The transformants with hygromycin resistance was identified by PCR and further confirmed by qRT-PCR with primers JD280-F/JD280-R (Table 3). In qRT-PCR assay, the tub2 was used as an internal control.

**Table 3:** Primers for construction and identification of *Fg*GCN5 gene deletion mutant

Primer	Sequence
280-1F	TAGCGTCTTCTCTTGATTGC
280-1R	TTGACCTCCACTAGCTCCAGCCAAGCCATGATTGGTGCGGGCTCAAC
280-2F	GAATAGAGTAGATGCCGACCGCGGGTTAACTAAAAGCGGGGAATCGG
280-2R	ACCAAGACCTATCACAGCAC
HYG-F	GGCTTGGCTGGAGCTAGTGGAGGTCAA
HY-R	GTATTGACCGATTCCTTGCGGTCCGAA
YG-F	GATGTAGGAGGGCGTGGATATGTCCT
HYG-R	AACCCGCGGTCGGCATCTACTCTATTC
JD280-F	TCGAAGAGCGCAATGGTG
JD280-R	TAGCGAATCCGTGGCAAC

### DON content measurement assay

DON content in LTB cultures [50, 51] was assayed with a competitive enzyme-linked immunosorbent assay (ELISA) based DON detection plate kit (Beacon Analytical Systems, Inc., USA) after incubation at 25 °C for 5 days, as described by Gardiner et al. [1]. In the assay, the *Tri5* deletion mutant was used as a negative control.

### Strains culture conditions

The wild-type strain PH-1 and the gene deletion mutant *Fggcn*5 of *F. graminearum* were cultured on potato dextrose agar (PDA) at 25 °C for 4 d. The mycelia were harvested and ground with MiniBead Beater-16 (Biospec, USA) at 30 Hz for 30 s. The ground mycelium were then cultured in 100 mL liquid YEPD medium with shaking at 25 °C for 24 h. Subsequently, the mycelium was collected by filtration with sterile macrocloth and was then incubated in DON inducing medium in the dark with shaking at 25 °C for 4 d. The DON inducing medium was prepared as described by Gardiner et al. [1].

### Protein extraction and trypsin digestion

The procedures of protein extraction and peptide digestion were modified from a previous report [15]. In brief, the mycelia samples were harvested and ground into cell powder in liquid nitrogen. The resulting cell powder was then transferred into 5 × volume of TCA/acetone (1:9, containing 65 mM DTT) and mixed by vortex. After placed at − 20 °C overnight, the mixture was centrifuged at 7000×g for 20 min at 4 °C. The precipitate was washed with ice-cold acetone for three times and was air-dried at 4 °C. The dried precipitate was then resolved in UA buffer (8 M urea, 150 mM Tris-HCl, Ph8.0) and sonicated 10 times (10 s burst with a 15 s interval for each time) on ice using a high intensity ultrasonic processor (Scientz, Ningbo, China). After centrifuged at 14,000 g for 40 min, the supernatant was filtered with 0.45 μm filters. The filtrate was quantified with the BCA Protein Assay Kit (Bio-Rad, USA).

The resulting protein solution was reduced with 10 mM DTT for 1 h at 37 °C and alkylated with 20 mM iodoacetamide for 45 min at room temperature in darkness. For trypsin digestion, 100 mM (NH_4_)_2_CO_3_ was added to urea concentration less than 2 M. Finally, trypsin was added at an enzyme-to-substrate mass ratio of 1:50 overnight and additional trypsin was added at an enzyme-to-substrate mass ratio of 1:100 for 4 h to ensure complete digestion.

### Immunoaffinity enrichment

The sample was separated into fractions by high pH reverse-phase HPLC followed Zhou et al. [15]. As a result, the tryptic peptides were separated into 6 fractions which were then dried by vacuum centrifuging.

The purification and enrichment of lysine acetylated peptides were performed as described [15, 52]. Briefly, tryptic peptides were re-dissolved in NETN buffer (100 mM NaCl, 1 mM EDTA, 50 mM Tris-HCl, 0.5% NP-40, pH 8.0). Subsequently, the pre-washed agarose-conjugated anti-acetyllysine antibody beads (Cat. No. 104, PTM Biolabs, Hangzhou, China) were added. The tryptic peptides were incubated at 4 °C overnight with gentle shaking. The beads were then washed four times with NETN buffer and twice with pure water. Finally, the bound peptides were eluted from the beads with 0.1% trifluoroacetic acid and were then vacuum-dried. The resulting peptides were cleaned with C18 ZipTips (Millipore, Billerica, MA) according to the manufacturer’s instructions before LC-MS/MS analysis.

### LC-MS/MS analysis

LC-MS/MS analysis was performed as described [53] with modification. In the assay, a Q-Exactive mass spectrometer (Thermo Scientific) coupled to Easy nLC (Proxeon Biosystems, now Thermo Fisher Scientific) was used. The mass spectrometer was operated in positive ion mode. MS data was acquired using a data-dependent top10 method dynamically choosing the most abundant precursor ions from the survey scan (300–1800 m/z) for HCD fragmentation. Automatic gain control (AGC) target was set to 3e^6^, and maximum inject time to 10 ms. Dynamic exclusion duration was 40.0 s. Survey scans were acquired at a resolution of 70,000 at m/z 200 and resolution for HCD spectra was set to 17,500 at m/z 200, and isolation width was 2 m/z. Normalized collision energy was 30 eV and the underfill ratio, which specifies the minimum percentage of the target value likely to be reached at maximum fill time, was defined as 0.1%. The instrument was run with peptide recognition mode enabled.

### Database search

The resulting MS/MS data was processed using MaxQuant with integrated Andromeda search engine (v.1.4.1.2). Tandem mass spectra were searched against UniProt_*F. graminearum* database concatenated with reverse decoy database. Trypsin was specified as the cleavage enzyme, the maximum missing cleavage was set as 4, and up to 5 modifications and 5 charges were allowed in each peptide. Mass error was set at 10 ppm for precursor ions and 0.02 Da for fragment ions. Carbamidomethylation on cysteine was specified as fixed modification and oxidation on methionine, acetylation on lysine and acetylation on protein N-terminal were specified as variable modifications. False discovery rate (FDR) thresholds for peptide and modification site were specified at 0.01. Minimum peptide length was set as 7. All the other parameters in MaxQuant were set to default values. The site localization probability was set as 0.75.

### Acetylated protein annotation

Gene Ontology (GO) annotation of identified acetylated proteins was derived from the UniProt-GOA database (http://www.ebi.ac.uk/GOA/). Firstly, the identified protein ID was converted to UniProt ID, and then mapped to GO ID by protein ID. The proteins were classified by GO annotation based on three categories: biological process, cellular component and molecular function. The subcellular localization of the protein was predicted with WoLF PSORT (http://wolfpsort.seq.cbrc.jp/). Secondary structures of proteins were predicted by NetSurfP [54]. Domain descriptions of identified protein were annotated by InterProScan5 based on protein sequence alignment, and the InterPro domain database (http://www.ebi.ac.uk/interpro/) was used. Kyoto Encyclopedia of Genes and Genomes (KEGG) database was used to annotate protein pathway [55]. Functional annotation tool of DAVID bioinformatics resources 6.7 was used to identify GO terms, KEGG pathways and protein domains [56].

### GO, KEGG pathway, domain and motif enrichment analysis

A two-tailed Fisher’s exact test was performed to examine the enrichment of the protein-acetylated entries against all proteins. Correction for multiple hypothesis testing was carried out using a previously described method [57]. Any term with a corrected *p* < 0.05 was considered significant.

### Acetylation protein–protein interaction network analysis

The protein–protein interaction was obtained from the STRING database following the methods [41, 25]. The protein–protein interaction network of the identified acetylated proteins was performed with Cytoscape software (version 3.2.1, www.cytoscape.org).

### Statistical analysis

Significant differences between the mutant and wild type strain were calculated according to the peptide intensity using a one-way analysis of variance with SPSS16.05 version. The *p* values of 0.05 were considered to be statistically significant.

## Supplementary information

**Additional file 1:** **Table S1.** The identified acetylated sites in Fggcn5 and PH-1.

**Additional file 2:** **Table S2.** The identified acetylated proteins in Fggcn5 and PH-1.

**Additional file 3:** **Table S3.** differentially acetylated proteins at significantly different level in Fggcn5 compared with PH-1.

**Additional file 4:** **Table S4.** The identified acetylated proteins specific in Fggcn5 or PH-1.

**Additional file 5:** **Table S5.** GO-based enrichment of the acetylated proteins.

**Additional file 6:** **Table S6.** KEGG pathway enrichment analysis.

**Additional file 7:** **Table S7.** Protein-protein interaction network of identified acetylated proteins.

## Data Availability

All data generated or analyzed during this study are included in this published article and its supplementary information files.

## Abbreviations

ABC: ATP-binding cassette
AGC: Automatic gain control
DON: Deoxynivalenol
DTT: Dithiothreitol
ELISA: Enzyme-linked immunosorbent assay
FDR: False discovery rate
FHB: Fusarium head blight
GO: Gene Ontology
HCD: Higher-energy collisionaldissociation
*Hph*: Hygromycin phosphotransferase
HPLC: High Performance Liquid Chromatography
KAT: Lysine acetyltransferase
KDAC: Lysine deacetylase
KEGG: Kyoto Encyclopedia of Genes and Genomes
PDA: Potato dextrose agar
PKA: cAMP- dependent protein kinase
PTM: Post-translational modification
TCA: Trichloroacetic acid
